# Proof-of-concept of a prior validated LC-MS/MS method for detection of *N*-lactoyl-phenylalanine in dried blood spots before, during and after a performance diagnostic test of junior squad triathletes

**DOI:** 10.3389/fspor.2025.1600714

**Published:** 2025-09-30

**Authors:** H. Bauhaus, T. Möller, S. Keller, A. Thomas, H. Braun, P. Wahl, M. Thevis

**Affiliations:** ^1^ Institute of Biochemistry, German Sport University Cologne, Cologne, Germany; ^2^ German Research Centre of Elite Sports, German Sport University Cologne, Cologne, Germany; ^3^ Centre for Preventive Doping Research, German Sport University Cologne, Cologne, Germany; ^4^ Institute of Exercise Science and Sport Informatics, Department of Exercise Physiology, German Sport University, Cologne, Germany; ^5^ Manfred Donike Institute for Doping Analysis, Cologne, Germany

**Keywords:** *N*-lactoyl-phenylalanine, appetite, metabolomics, dried blood spots, RED-S, biomarker, athletes, endurance exercise

## Abstract

**Introduction:**

*N*-lactoyl-phenylalanine (lac-phe) is an exercise-inducible metabolite that is discussed for suppressing appetite. As no point-of-care lac-phe analysis for frequent sample collection during exercise exists, the objectives of this study were (1) to develop and validate a method for quantifying lac-phe by liquid chromatography tandem mass spectrometry (LC-MS/MS) using 20 µl of capillary blood collected on cellulose-based dried blood spot (dbs) cards (QIAcard® FTA® DMPK-C) and (2) to determine lac-phe dbs concentrations before, during, and after a combined running and cycling performance test protocol in regional junior squad triathletes for proof-of-concept.

**Methods:**

The validated method (precision <8%, accuracy <7% in a working range of 0–100 ng/ml, stability >28 days at −20°C and 20°C) was successfully applied in a combined running and cycling performance diagnostic test. Dbs samples were collected by trained researchers at multiple time points – before, during, and after the performance diagnostic test - from 15 female and male junior squad triathletes on two test occasions separated by 3 months and a training period.

**Results:**

Lac-phe concentrations increased from 31.5 ± 10 nmol/L at baseline to a maximum of 101.0 ± 40.1 nmol/L at time-to-exhaustion after the second incremental test. 15 min after exercise, lac-phe concentration decreased to 89.7 ± 30.4 nmol/L. No statistical difference between the two test occasions was seen. Sex-specific differences were observed, with female subjects showing significantly higher lac-phe concentrations than male subjects (*p* < 0.05) at time-to-exhaustion after the first incremental test and at the onset of the second incremental test. No statistically significant correlations were identified between lac-phe and lactate, glucose as well as anthropometric and respiratory parameters.

**Conclusion:**

This study introduces a robust method that, for the first time, enables the quantification of lac-phe concentrations not only before and after, but also during a performance test. By applying this approach, it contributes novel insights into the temporal dynamics of exercise-induced alterations in lac-phe concentrations in humans.

## 1 Introduction

Physical activity stimulates various metabolic, cardiovascular, and immunological signaling pathways, most of which have beneficial effects on overall health, and interactions are being investigated employing multi-omics approaches, such as proteomics, transcriptomics, genomics, and metabolomics (1, 2). The development and improvement of detection methods for new metabolites to assess health status are and will be increasingly important in terms of personalized nutrition and training (2, 3).

While performing untargeted and targeted metabolomic analysis in *in vitro* media, several *N*-lactoyl-amino acids were discovered. In the presence of lactate, the highest elevation in *N*-lactoyl-amino acids was found for *N*-lactoyl-phenylalanine (lac-phe) (C_12_H_15_NO_4,_ average molecular mass 237.255 Da). Lac-phe is a lactoyl derivative of phenylalanine, a pseudodipeptide formed from lactate and phenylalanine by reverse proteolysis catalyzed by the enzyme carnosine dipeptidase 2 (CNDP2). The plasma levels of lac-phe strongly correlate with the plasma levels of lactate and phenylalanine. Since lactate, phenylalanine, and CNDP2 are ubiquitous, *N*-lactoyl-amino acids are present in many tissues (4). Simultaneously, improved glucose homeostasis, reduced energy intake, and weight loss were found when injection-induced lac-phe levels were elevated in mice. In contrast, when implementing an exercise program, CNDP2-knock out (KO) mice had a higher food intake and reduced body weight loss compared to control mice (5).

First data in healthy, moderately trained and normal-weight (BMI: 23.1 ± 2.1 kg/m, $\dot{V}\;O_{2}$max: 42.6 ± 4.2 ml/kg/min) humans has shown an increase of plasma lac-phe concentrations after performing a 3 × 30 s sprint protocol from immediately pre- to 60 min after the exercise (5). Another study found a decrease in subcutaneous fat tissue that has been shown to correlate with lac-phe plasma concentration after implementing a training intervention in sedentary and obese human subjects with diabetes mellitus type 2. Therefore, a therapeutic potential of lac-phe is discussed for patients suffering from obesity and/or diabetes mellitus and for predicting the success of a training program aiming for fat loss (6). As previous studies were performed *in vitro*, in animals, obese or moderately trained humans (4–6), lac-phe dynamics in lean, healthy, and well-trained individuals has not been explored, yet. A group, vulnerable to negative energy balance are athletes. Due to their elevated energy expenditure associated with training and competition, have substantially higher energy requirements than non-athletes. However, maintaining energy balance can be challenging in this population, while the reasons for this are still under research. In a consequence, many athletes experience low energy availability (LEA) (7). Chronic LEA has been linked to adverse effects on health and performance referred to as relative energy deficiency in sports (RED-S) (8). A metabolic-mediated link between exercise and appetite suppression – as has been hypothesized for lac-phe – could offer an additional explanatory mechanism underlying LEA and RED-S in athletes (9, 10). However, enhanced lactate turnover particularly seen in well trained endurance athletes may influence the kinetics of lactate-derived metabolites like lac-phe (11, 12).

Therefore, within the scope of this study, lac-phe concentrations should be quantified in trained regional youth squad triathletes before, during, and after a metabolically demanding combined running and cycling performance diagnostic test. As no point-of-care diagnostic tool exists that allows for practical lac-phe analysis during exercise, an analytical method based on dried blood spot (dbs) sampling combined with liquid chromatography tandem mass spectrometry (LC-MS/MS) has been developed and validated prior to the present investigation (Supplementary Material). In the human metabolite database, lac-phe (MDB0062175) is labelled as “detected but not quantified”, highlighting the need for reliable quantification strategies. Therefore, the aims of this study were twofold: (1) to validate a dbs-based method for lac-phe using d_5_-lac-phe as an internal standard (ISTD) for quantification and sampling during exercise and (2) to apply this method in order to determine endogenous dbs lac-phe concentrations in regional junior squad triathletes during a performance test, thereby assessing whether previously reported exercise-induced increases, observed in animal models and in untrained and/or obese human, can be replicated in a lean, well-trained athletic cohort.

## 2 Methods

### 2.1 LC-MS/MS method development and validation

Analytical method validation was performed following the main requirements of the U.S. Food and Drug Administration (FDA) guidelines for bioanalytical method validation (13). These include parameters like linearity, precision, accuracy, carryover, matrix effects, recovery, selectivity, stability, limit of detection (LOD) and limit of quantification (LOQ). For precise description on the method development including synthesis of the internal standard (14) and validation process, see Supplementary Material.

### 2.2 Subjects

Fifteen male and female triathletes (*n* = 15, nine males and six females) from the North Rhine Westphalian (regional) triathlon youth squad were included in the study (*mean + sd*, age: 16 ± 2 years, bodyweight: 60 ± 10 kg, height: 171 ± 8 cm, $\dot{V}\;O_{2}$max: 59 ± 6 ml/min/kg) and tested on two occasions (Table 1 for sex-specific and occasion-specific data). Two subjects dropped out of the post-measurement period. One subject, that showed implausible lac-phe data due to a methodological error, was excluded from the analysis. The participants and their legal guardians were informed about the benefits and risks of the study and provided written informed consent prior to participation. The study was approved and based on the Declaration of Helsinki by the university's local ethics committee (011/2023) before the start of data collection.

**Table 1:** Anthropometric data of subjects displayed sex-specific and for each test occasion pre and post.

Sample	Age (years)	Weight (kg)	Height (cm)	BMI (kg/m^2^)	FFM (kg)	FM (kg)	BFP (%)	$\dot{V}\;O_{2}$max (ml/kg/min)
Male_pre_ = 9	16 ± 2	63 ± 11	175 ± 8	21 ± 2	58 ± 8	6 ± 3	10 ± 4	63 ± 4
Male_post_ = 8	16 ± 2	63 ± 10	174 ± 7	21 ± 2	57 ± 7	5 ± 4	9 ± 4	62 ± 4
Female_pre_ = 6	17 ± 2	55 ± 6	165 ± 5	20 ± 2	45 ± 3	10 ± 3	18 ± 4	54 ± 6
Female_post_ = 5	17 ± 2	55 ± 6	165 ± 4	20 ± 2	45 ± 3	10 ± 4	18 ± 5	55 ± 6

### 2.3 Study procedure

The subjects arrived at the laboratory on two different occasions, separated by a preparatory training phase prior to the season between their two test days. Subjects were educated on maintaining an adequate carbohydrate (CHO) intake of 5–8 g/kg body weight (BW) in the two days before the test day and consumed their habitual breakfast and lunch on the test day prior to the exercise protocol. The participants were asked to report their food intake and exercise activity using a validated reporting protocol (15). Before starting the test, bioimpedance analysis was performed to determine the body composition (SECA Medical Body Composition Analyzer, Hamburg, Germany). On both test days, the subjects performed their tests at the same time in the afternoon. During the diagnostic test, the subjects drank water *ad libitum* but did not consume any energy-containing foods or beverages.

### 2.4 Performance diagnostic protocol

**Figure 1:**
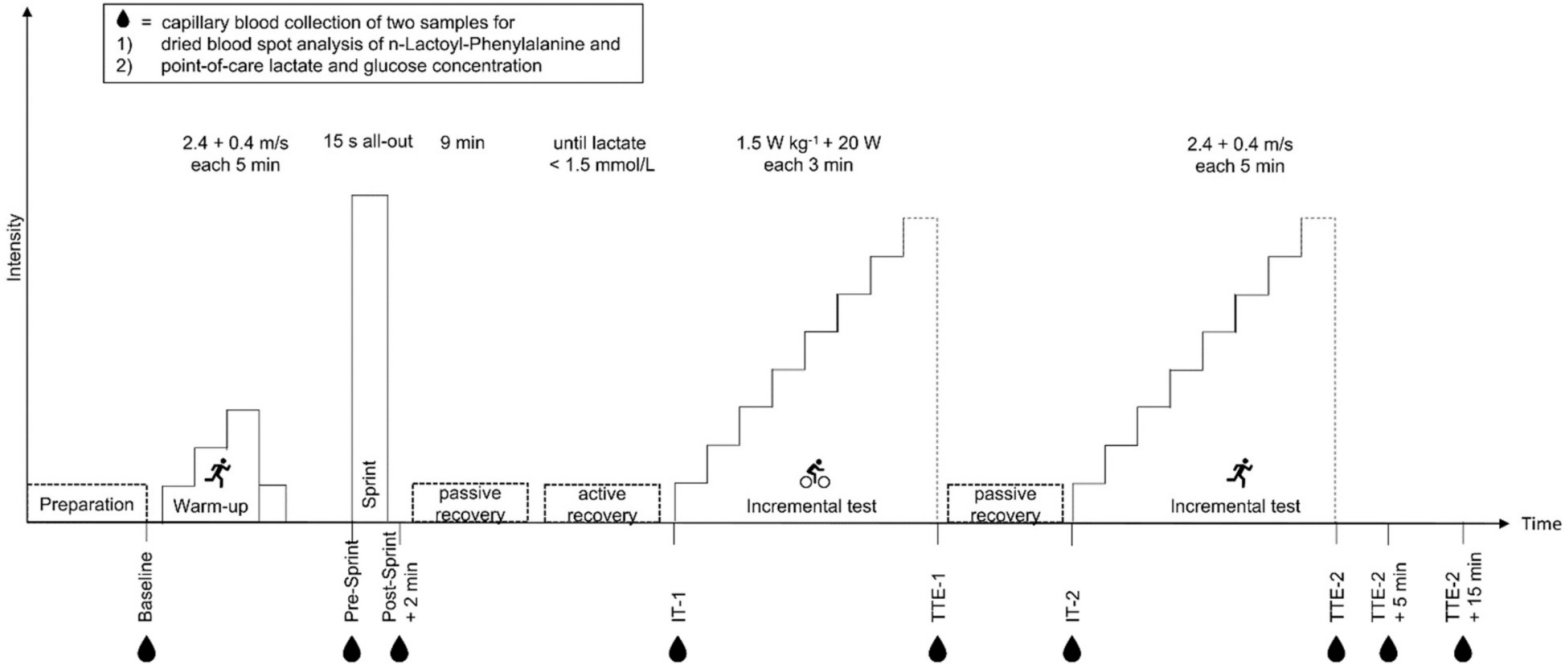
Study procedure, timepoints for blood sample collection: baseline: baseline measurement before the start of the test in resting supine position, Pre-sprint: before a 15 s all-out sprint, post-sprint + 2 min: 2 min after the sprint, IT-1: at start of the first incremental test after 9 min of passive recovery and subsequent active recovery until lactate concentration was <1.5 mmol/L, TTE-1: at time-to-exhaustion after the first incremental test, IT-2: at start of the second incremental test after 8 min of passive recovery, TTE-2: at time-to-exhaustion after the second incremental test,TTE-2 + 5 min: 5 min after the second incremental test at rest, TTE-2 + 15 min: 15 min after the second incremental test at rest.

Data were collected before, during and after the diagnostic test (Figure 1). The comprehensive test protocol has been described elsewhere (16). Concerning this study, in brief, the first blood sample was collected before the test for a baseline (BL) in a seated and resting position. After the warm-up (running at 2.4 m/s + 0.4 m/s every five minutes for 15 min) on a treadmill (h/p/cosmos pulsar® 3p, Traunstein, Germany), a 15 s all-out sprint-test on a bike ergometer (SRM ErgoMeter, Juelich, Germany) followed. Blood samples were taken before (Pre-Sprint) and two minutes after the test (Post-Sprint +2 min). After 9 min of passive recovery, active recovery was performed until lactate concentration decreased below 1.5 mmol/L (10–15 min). Afterwards, a first incremental step test on a bike ergometer (1.5 W/kg + 20 W every three minutes) was performed till volitional exhaustion. Blood samples were collected before (IT-1) and at time-to-exhaustion (TTE-1). A second incremental step test on the treadmill followed and was also carried out until exhaustion. Blood samples were taken before (IT-2), directly after (TTE-2) as well as five (TTE-2 + 5 min) and 15 min (TTE-2 + 15 min) after the test. The incremental bike and treadmill tests were separated by 8 min of passive rest. Spirometric data (Metalyzer 3B Cortex Biophysik GmbH, Leipzig, Germany) were collected during the warm-up and both incremental tests every second. The spirometer was calibrated on each testing day before the first test with a reference gas and before each test section with ambient air and a 3 L-syringe according to the manufacturer's instructions.

### 2.5 Blood sampling and analysis

Capillary blood samples were collected from the earlobe by trained researchers using a 20 µl capillary tube. The tube was emptied on a dbs filter card (QIAcard FTA DMPK-C) for lac-phe analysis. Simultaneously, using another 20 µl capillary tube, capillary lactate and glucose samples were taken and directly analyzed using Biosen C-Line (EKF diagnostics Holding, Cardiff, UK). The dbs cards were dried for at least three hours at room temperature in the dark. Subsequently, the dbs cards were stored individually in Ziploc plastic bags with a desiccant (silica gel) at −80°C until analysis. The detailed sample preparation is explained in the Supplementary Material. Briefly, the entire dried blood spot was punched from the card, cut, and extracted into 600 µl of methanol (100%) in a tube. To quantify lac-phe, an internal standard (d_5_-lactoyl-phenylalanine) was added to the tubes. After sonication for 30 min, the tubes were centrifuged at 17,000 × g for 10 min (19°C). The supernatant was dried in a vacuum centrifuge for 90 min (45°C) and reconstituted with a 10% acetonitrile solution. After a second centrifugation at 17,000 × g for 10 min (19°C), the supernatant was transferred into a vial and analyzed using LC-MS/MS (for instrumental conditions, please see Supplementary Material). Lac-phe concentrations exceeding the validated linear working range of 0–100 ng/ml were calculated by extrapolation.

### 2.6 Statistical analysis

Statistical data analysis was conducted using R version 4.1.3 (The R Foundation for Statistical Computing) and Excel 2019 (Microsoft Corp., Redmond, WA, USA). Data are presented as mean ± standard deviation. Coefficients of variations were calculated. Before performing parametric or non-parametric tests, normality of residuals was tested using the Shapiro–Wilk test and graphical analysis by QQ plots and histograms. Wilcoxon sum rank test or independent *t*-test for independent samples and Wilcoxon or paired *t*-test was performed for dependent samples. To quantify the magnitude of changes between pre vs. post occasion and male vs. female subjects, respectively, Cohen's effect sizes (ESs) were calculated and classified as trivial (ES <0.2), small (0.2 ≤ ES <0.6), moderate (0.6 ≤ ES <1.2), and large (1.2 ≤ ES <2.0) (17). Since some participants completed two tests at different times (pre and post), associations between variables were assessed using mixed linear models with the independent variable as a fixed effect and a random intercept for participant ID to account for repeated measurements. Estimated slopes were classified as trivial (|r| < 0.1), low (0.1 ≤ |r| <µ 0.3), moderate (0.3 ≤ |r| < 0.5), high (0.5 ≤ |r| < 0.7), very high (0.7 ≤ |r| < 0.9), and nearly perfect (|r| ≥ 0.9) (17). Only data within the 99% confidence interval were considered in the final statistical analysis, which led to exclusion of three values above this interval.

## 3 Results

### 3.1 Validation of the LC-MS/MS method

A summary of all the validation results is presented in the Supplementary Material (Supplementary Table S1).

### 3.2 Nutritional data

There was no statistical significant difference in subjects' nutrition within the two days before the first (pre) and the second (post) diagnostic, considering energy intake (pre 2,582.7 ± 1,180.2 kcal/day, post 2,639.8 ± 1,230.7 kcal/day; *p* = 0.727), relative energy intake (pre 44.2 ± 16.6 kcal/kg BW/day, post 43.8 ± 16.9 g/kg BW/day; *p* = 0.844), CHO intake (pre 335.3 ± 151.6 g/day, post 341.2 ± 136.4 g/day; *p* = 0.78) and relative CHO intake (pre 5.8 ± 2.2 g/kg BW/day, post 5.7 ± 2.1 g/kg BW/day; *p* = 0.972).

### 3.3 Glucose, lactate and Lac-Phe data

Throughout the performance test and subsequent recovery period of 15 min, the mean capillary glucose concentration was 4.9 ± 0.8 mmol/L, the capillary lactate concentration was 4.6 ± 3.3 mmol/L, and the dbs lac-phe concentration in dried whole-blood spot samples was 67.9 ± 37.3 nmol/L. Individual curves are displayed in the Supplementary Material (Supplementary Figures S1a–c). Individual maximum dbs lac-phe concentrations were reached before, immediately after, and five minutes after the second incremental test (IT-2: n_max_ = 11, TTE-2: n_max_ = 9, TTE-2 + 5 min: n_max_ = 8). The mean maximum dbs lac-phe concentration was 107 ± 37 nmol/L.

### 3.4 Pre and post tests

**Figure 2:**
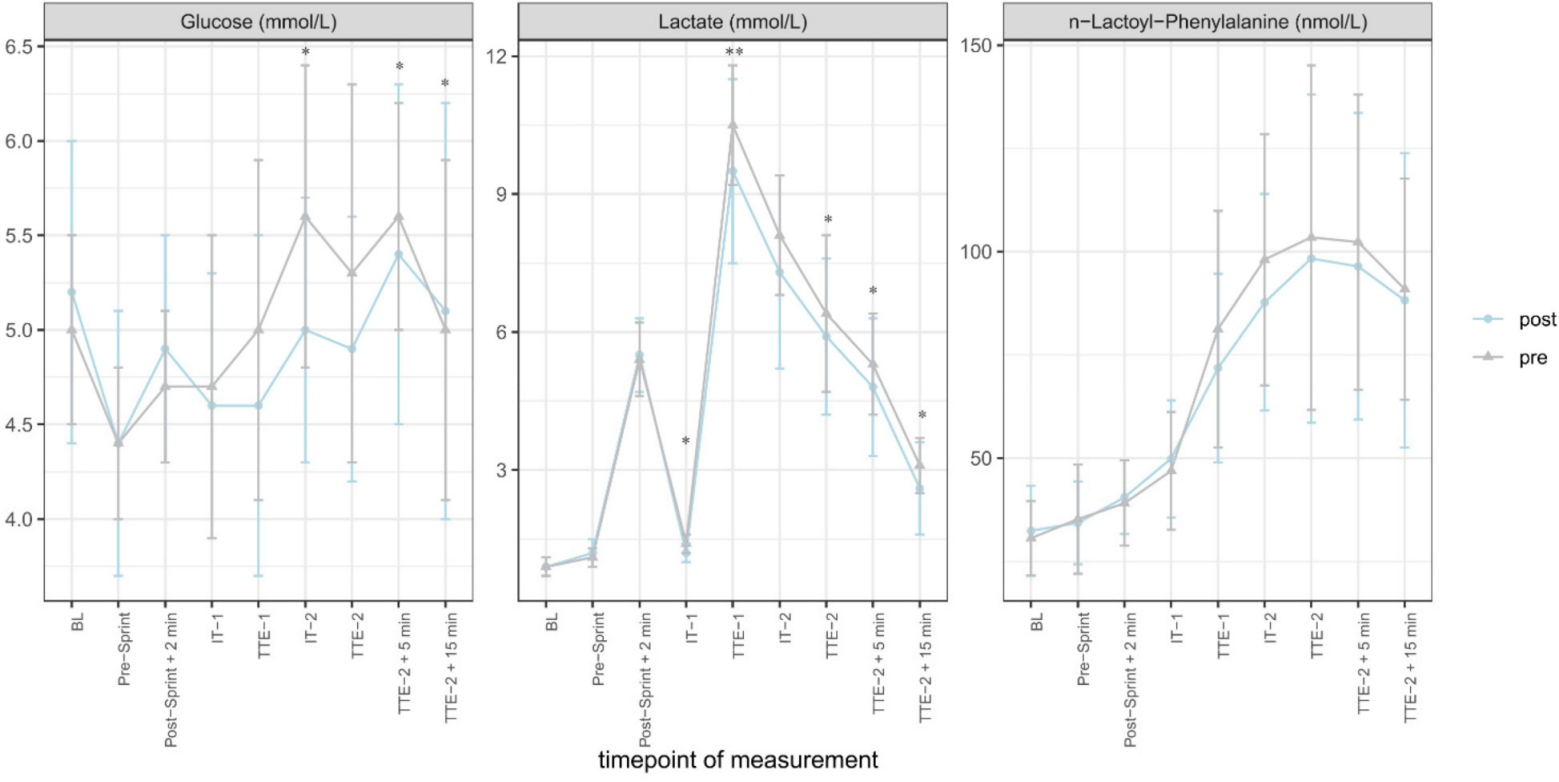
Capillary glucose, capillary lactate and dried blood spot (dbs) *N*-lactoyl-phenylalanine (lac-phe) concentrations over the course of the study differentiated by test occasion and shown as mean ± standard deviation; *p < 0.05, **p < 0.001. (grey line = pre, blue line = post).

Capillary glucose concentrations were significantly lower at IT-2 (*p* = 0.043) and TTE-2 + 5 min (*p* = 0.017) but significantly higher at TTE-2 + 15 min (*p* = 0.014) in the post-test than in the pre-test. Capillary lactate concentrations were significantly lower for IT-1 (*p* = 0.028), TTE-1 (*p* = 0.004), TTE-2 (*p* = 0.021), TTE-2 + 5 min (*p* = 0.021), and TTE-2 + 15 min (*p* = 0.025) in the post-test than in the pre-test. Dbs lac-phe concentrations did not show significant differences for any timepoint when comparing pre- and post-test (Figure 2). The mean concentrations, standard deviations, coefficients of variation coefficients, and statistical p-values for each timepoint are displayed in Table 2.

**Table 2:** Mean capillary glucose, capillary lactate, and dried blood spot (dbs) *N*-lactoyl-phenylalanine (lac-phe) concentrations, standard deviation, and coefficient of variation for each timepoint of the protocol differentiated by test occasion; statistical significance between pre- and post-test concentrations is calculated by paired parametric or non-parametric test and indicated by *p* < 0.05 and effect size calculated as Cohen's d for parametric tests and r for non-parametric tests.

Timing		Glucose (mmol/L)			Lactate (mmol/L)			*N*-Lac-Phe (nmol/L)		
		M ± SD	P r/d	CV	M ± SD	P r	CV	M ± SD	P r/d	CV
BL	Pre	5.0 ± 0.5	p: .402 r: .233	10.4	0.9 ± 0.2	p: .625 r: .136	20.5	30.6 ± 9.0	p: .675 r: .116	29.5
	Post	5.2 ± 0.8		15.5	0.9 ± 0.2		23.9	32.4 ± 10.9		33.7
Pre-Sprint	Pre	4.4 ± 0.4	p: .755 d: .089	9.7	1.1 ± 0.2	p: .861 r: .048	21.3	35.2 ± 13.2	p: .967 d: .012	37.6
	Post	4.4 ± 0.7		15.3	1.2 ± 0.3		29.0	34.3 ± 10.0		29.1
Post-Sprint + 2 min	Pre	4.7 ± 0.4	p: .173 r: .387	9.2	5.4 ± 0.8	p: 1 r: 0	15.6	39.1 ± 10.3	p: .358 d: .265	26.5
	Post	4.9 ± 0.6		12.7	5.5 ± 0.8		14.4	40.5 ± 8.9		21.9
IT-1	Pre	4.7 ± 0.8	p: .727 r: .097	17.5	**1.4** ± **0.2**	**p: .028** **r: .611**	15.1	46.9 ± 14.2	p: .108 r: .446	30.2
	Post	4.6 ± 0.7		15.5	**1.2** **±** 0.2*		14.3	49.8 ± 14.2		28.5
TTE-1	Pre	5.0 ± 0.9	p: .162 r: .388	17.9	**10.5** ± **1.3**	**p: .004** **r: .795**	12.3	81.2 ± 28.6	p: .675 r: .116	35.2
	Post	4.6 ± 0.9		19.3	**9.5** **±** 2.0**		21.1	71.8 ± 22.8		31.8
IT-2	Pre	**5.6** **± 0.8**	**p: .043** **r: .562**	13.9	8.1 ± 1.3	p: .069 r: .504	15.8	98.0 ± 30.5	p: .889 r: .039	31.1
	Post	**5.0** ± 0.7*		14.7	7.3 ± 2.1		28.7	87.7 ± 26.2		29.9
TTE-2	Pre	5.3 ± 1.0	p: .364 r: .252	19.7	**6.4** **± 1.7**	**p: .021** **r: .349**	27.1	103.4 ± 41.7	p: 1 r: 0	40.3
	Post	4.9 ± 0.7		13.4	**5.9** ± 1.7*		29.0	98.3 ± 39.8		40.5
TTE-2 + 5 min	Pre	**5.6** **± 0.6**	**p: .017** **r: .664**	11.3	**5.3** **± 1.1**	**p: .021** **r: .642**	21.1	102.3 ± 35.8	p: 1 r: 0	35.0
	Post	**5.4** ± 0.9*		17.5	**4.8** ± 1.5*		32.1	96.4 ± 37.1		38.5
TTE-2 + 15 min	Pre	**5.0** **± 0.9**	**p: .014** **r: .685**	17.2	**3.1** **± 0.6**	**p: .025** **r: .62**	19.3	90.9 ± 26.8	p: .561 d: .166	29.4
	Post	**5.1** ± 1.1*		22.5	**2.6** ± 1.0*		36.3	88.2 ± 35.6		40.4

*p < 0.05; **p < 0.001.

### 3.5 Analysis for sex

**Figure 3:**
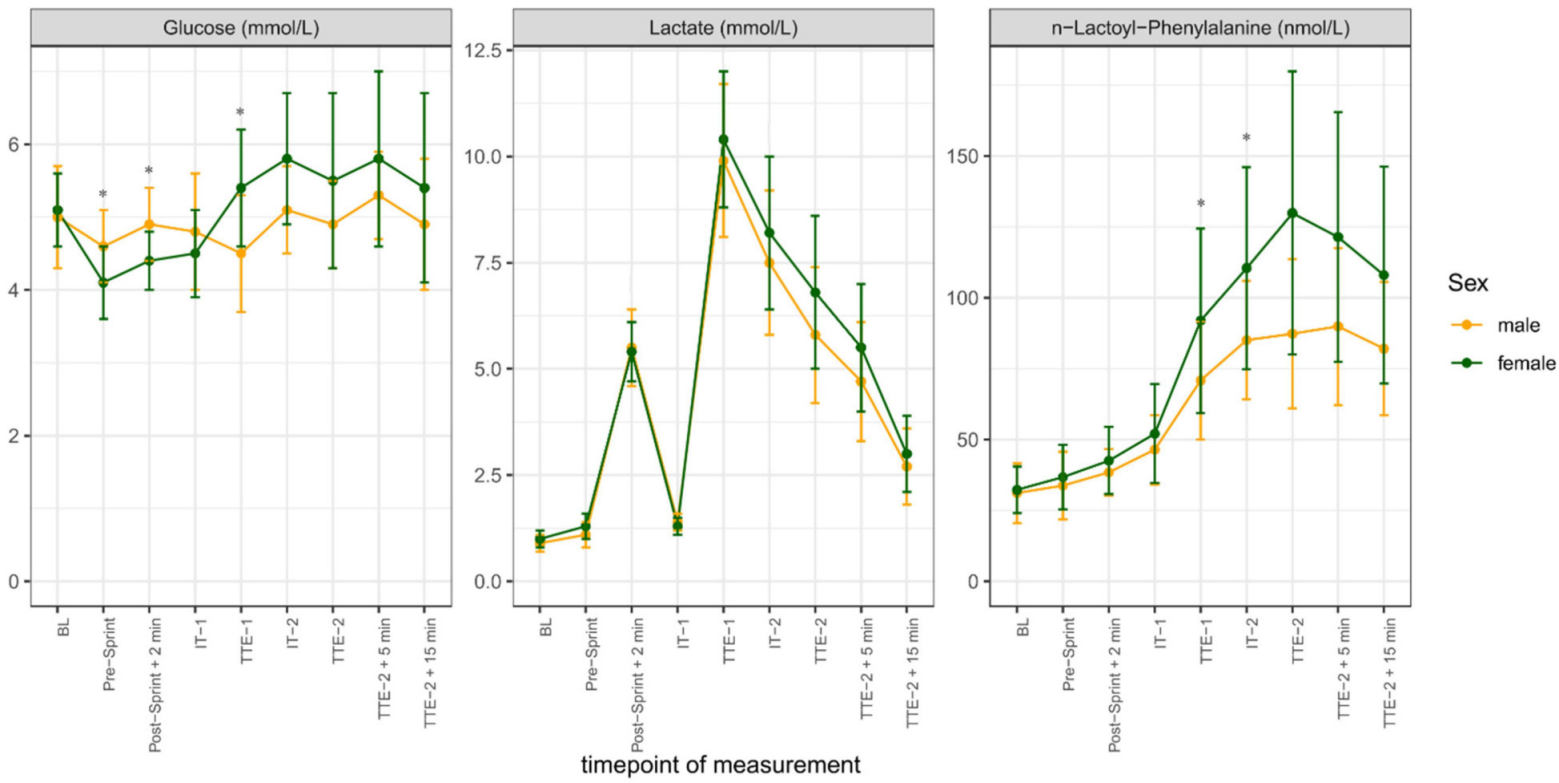
Capillary glucose, capillary lactate and dried blood spot (dbs) *N*-lactoyl-phenylalanine (lac-phe) concentrations over the course of the study differentiated by sex and shown as mean ± standard deviation; *p < 0.05, **p < 0.001. (yellow line = male, green line = female).

Capillary glucose concentration was significantly higher at TTE-1 (*p* = 0.006) and at IT-2 (*p* = 0.044) and lower at Pre-Sprint (*p* = 0.021) and at Post-Sprint + 2 min (*p* = 0.032) in females than in males. No significant differences in capillary lactate concentrations were found between sexes. Dbs lac-phe concentrations at IT-2 (*p* = 0.041) and TTE-2 (*p* = 0.015) were significantly higher in female subjects. In general, mean female capillary lactate and dbs lac-phe concentrations tended to be higher than mean male concentrations, especially during incremental tests (Figure 3). The mean concentrations, standard deviations, coefficients of variation, and statistical p-values for each timepoint are displayed in Table 3.

**Table 3:** Mean capillary glucose, capillary lactate, and dried blood spot (dbs) *N*-lactoyl-phenylalanine (lac-phe) concentrations, standard deviation, and coefficient of variation for each timepoint of the protocol differentiated by sex, statistical significance between sex-related concentrations is calculated by independent parametric or non-parametric test and indicated by *p* < 0.05 and effect size calculated as Cohen's r for non-parametric tests.

Timing		Glucose (mmol/L)			Lactate (mmol/L)			*N*-Lac-Phe (nmol/L)		
		M ± SD	P r	CV	M ± SD	P r	CV	M ± SD	P r	CV
BL	M	5.0 ± 0.7	p: .640 r: .088	14.5	0.9 ± 0.2	p: .184 r: .251	21.6	31.1 ± 10.6	p: .507 r: .126	34.3
	F	5.1 ± 0.5		10.1	1.0 ± 0.2		21.4	32.2 ± 8.2		25.6
Pre-Sprint	M	**4.6** **± 0.5**	**p: .021** **r: .437**	10.6	1.1 ± 0.3	p: .201 r: .242	23.4	33.8 ± 11.9	p: .363 r: .172	35.2
	F	**4.1** ± 0.5*		12.9	1.3 ± 0.3		25.1	36.8 ± 11.4		31.1
Post-Sprint + 2 min	M	**4.9** **± 0.5**	**p: .032** **r: .404**	10.8	5.5 ± 0.9	p: .883 r: .028	15.9	38.4 ± 8.2	p: .363 r: .172	21.5
	F	**4.4** ± 0.4*		8.4	5.4 ± 0.7		12.4	42.6 ± 11.9		27.8
IT-1	M	4.8 ± 0.8	p: .446 r: .144	17.4	1.4 ± 0.2	p: .279 r: .205	17.1	46.4 ± 12.2	p: .476 r: .135	26.2
	F	4.5 ± 0.6		13.7	1.3 ± 0.2		14.2	52.1 ± 17.4		33.3
TTE-1	M	**4.4** **± 0.8**	**p: .006** **r: .521**	17.8	9.9 ± 1.8	P: .980 R: .005	17.9	70.8 ± 20.8	p: .228 r: .228	29.4
	F	**5.4** ± 0.8*		14.6	10.4 ± 1.6		15.5	91.9 ± 32.6		35.4
IT-2	M	**5.1** **± 0.6**	**p: .044** **r: .381**	12.0	7.5 ± 1.7	P: .941 R: .014	22.6	**85.0** **± 20.9**	**p: .041** **r: .386**	24.6
	F	**5.8** ± 0.9*		16.2	8.2 ± 1.8		22.1	**110.5** ± 35.6*		32.2
TTE-2	M	4.9 ± 0.6	p: .313 r: .191	12.8	5.8 ± 1.6	p.279 r: .205	27.7	**87.3** **± 26.3**	**p: .015** **r: .46**	30.1
	F	5.5 ± 1.2		22.1	6.8 ± 1.8		26.4	**129.9** ± 49.9*		38.5
TTE-2 + 5 min	M	5.3 ± 0.6	p: .939 r: .014	12.0	4.7 ± 1.4	p: .819 r: .042	29.1	89.9 ± 27.7	p: .105 r: .307	30.8
	F	5.8 ± 1.2		20.9	5.5 ± 1.5		27.4	121.4 ± 44.0		36.3
TTE-2 + 15 min	M	4.9 ± 0.9	p: .939 r: .014	18.9	2.7 ± 0.9	p: .98 r: .005	33.6	82.0 ± 23.5	p: .134 r: .284	28.7
	F	5.4 ± 1.3		24.6	3.0 ± 0.9		28.3	108.0 ± 38.3		35.5

*p < 0.05.

### 3.6 Linear mixed model

**Figure 4:**
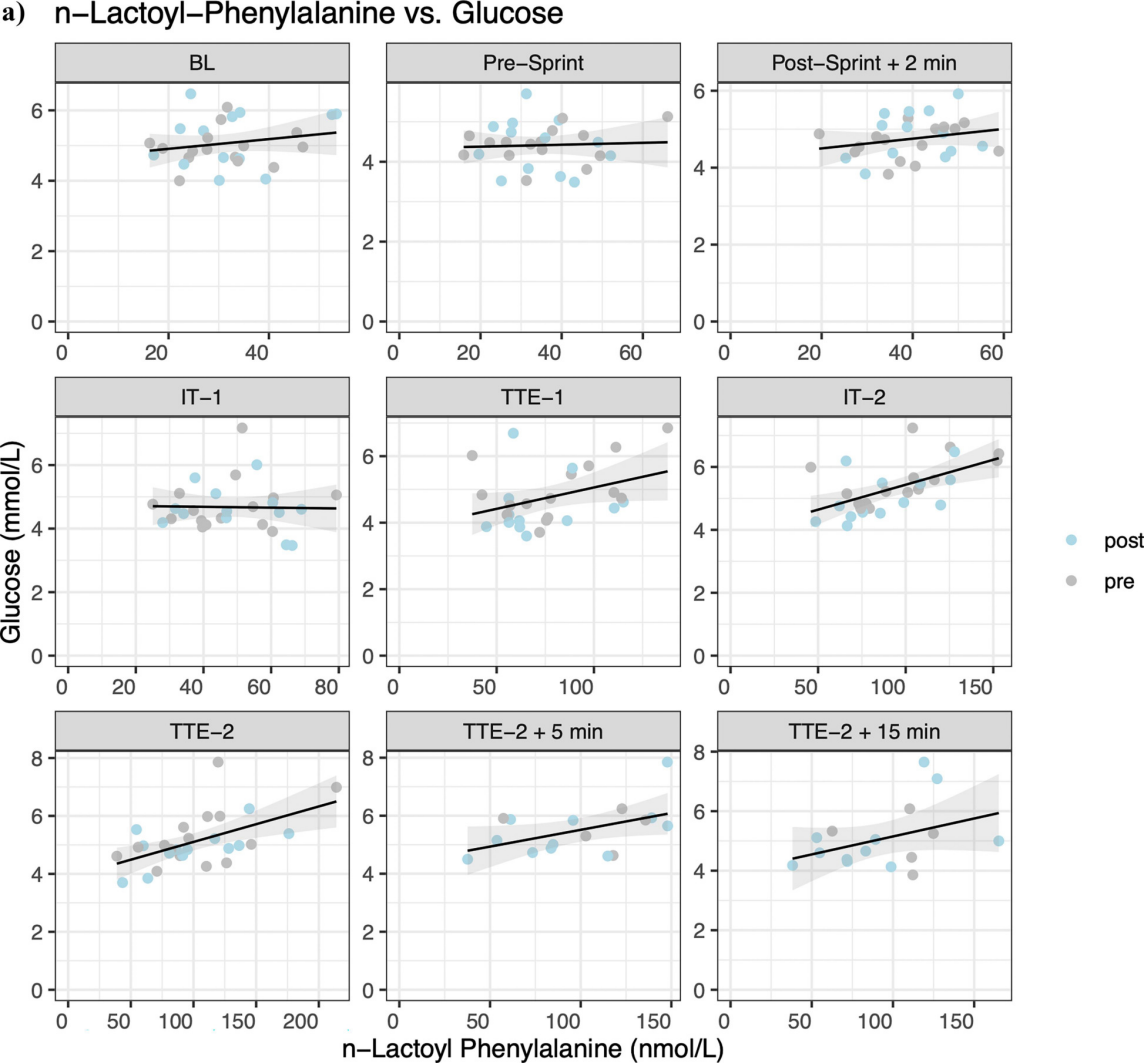
Correlation of **(a)** *N*-lactoyl-phenylalanine (lac-phe) and glucose, **(b)** lac-phe and lactate and **(c)** cross-correlation of lac-phe at timepoint t + 1 and lactate at timepoint t.

Linear mixed effect models indicated statistical significance between lac-phe and glucose concentrations at IT-2 (*β* = 0.016, *p* = 0.002), TTE-2 (*β* = 0.012, *p* = 0.003), TTE-2 5 min (*β* = 0.011, *p* = 0.032), and TTE-2 15 min (*β* = 0.015, *p* = 0.025) (Figure 4A). For lac-phe and lactate concentrations, statistical significance is indicated at Post-Sprint + 2 min (*β* = 0.016, *p* = 0.034), TTE-1 (*β* = 0.027, *p* = 0.032), IT-2 (*β* = 0.030, *p* = 0.010), TTE-2 (*β* = 0.018, *p* = 0.006), and TTE-2 15 min (*β* = 0.013, *p* = 0.047) (Figure 4B). For lactate concentration at timepoint t and lac-phe concentration at timepoint t + 1, statistical significance is indicated at TTE-1 (*β* = 0.027, *p* = 0.016), IT-2 (*β* = 0.016, *p* = 0.048), TTE-2 (*β* = 0.022, *p* = 0.007) and TTE-2 + 5 min (*β* = 0.021, *p* = 0.045) (Figure 4C). Although statistically significant, the estimated slopes indicate only weak associations. Non-significant results were found for the AUC (t4-t5) of lac-phe and the AUC of lactate (t5-t4) (*β* = 0.002, *p* = 0.766), and time to exhaustion (*β* = 0.712 *p* = 0.169), as well as for the AUC (t4-t7) of lac-phe and the AUC (t4-t7) of lactate (*β* = 0.016, *p* = 0.082), the accumulated time to exhaustion (*β* = 0.954, *p* = 0.302), CHO oxidation (*β* = 0.036, *p* = 0.700), fat oxidation (*β* = −0.007, *p* = 0.594), energy expenditure (EEx) (*β* = 0.196, *p* = 0.552), $\dot{V}\;O_{2}$max (*β* = 0.005, *p* = 0.557), body mass index (BMI) (*β* = 0.001, *p* = 0.440), body fat percentage (BFP) (*β* = 0.007, *p* = 0.434), muscle mass (MM) (*β* = −0.007, *p* = 0.191), fat mass (FM) (*β* = 0.003, *p* = 0.672), fat-free mass (FFM) (*β* = 0.000, *p* = 0.949) and bodyweight (BW) (*β* = 0.001, *p* = 0.831).

**Figure:**
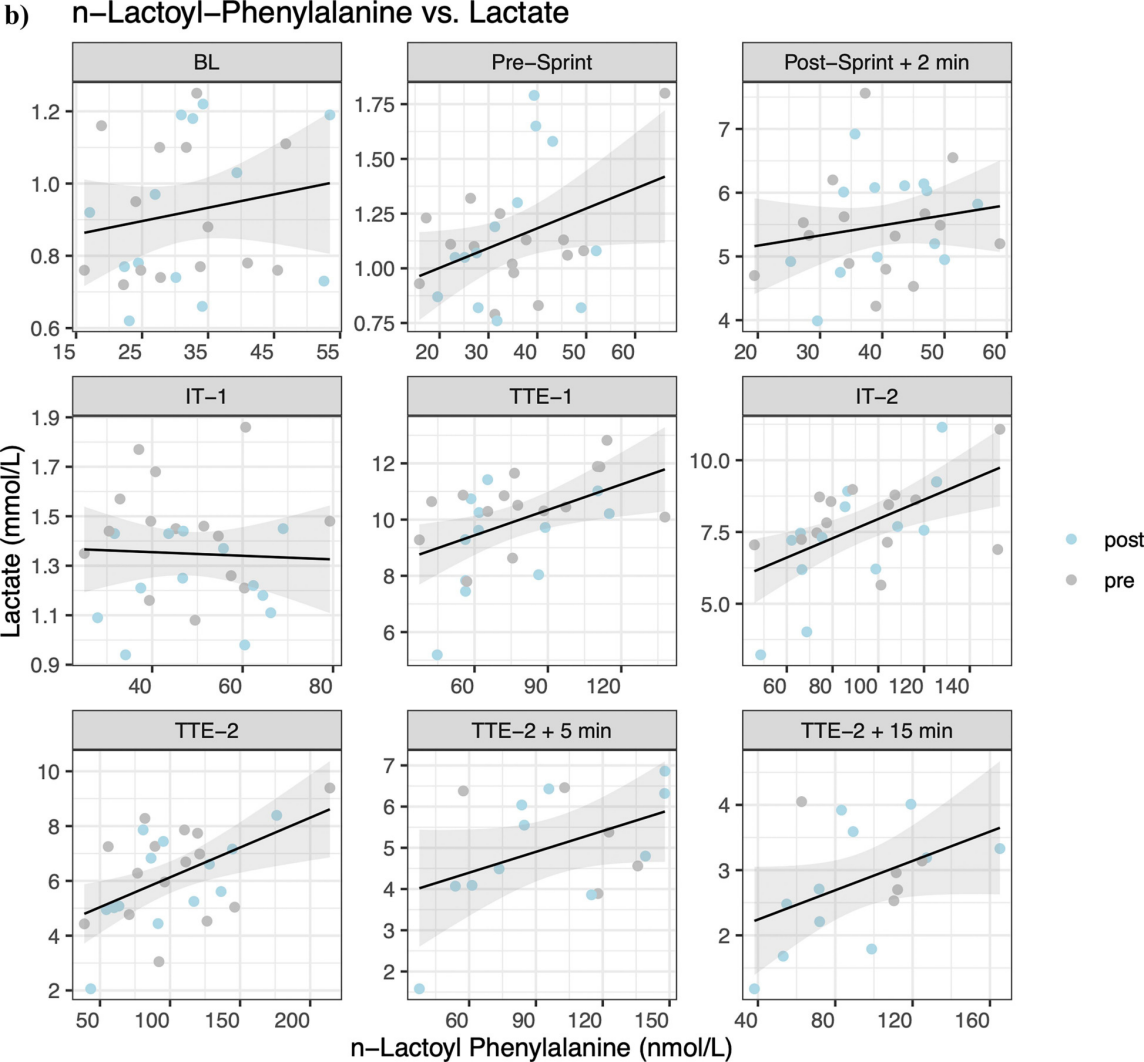


**Figure:**
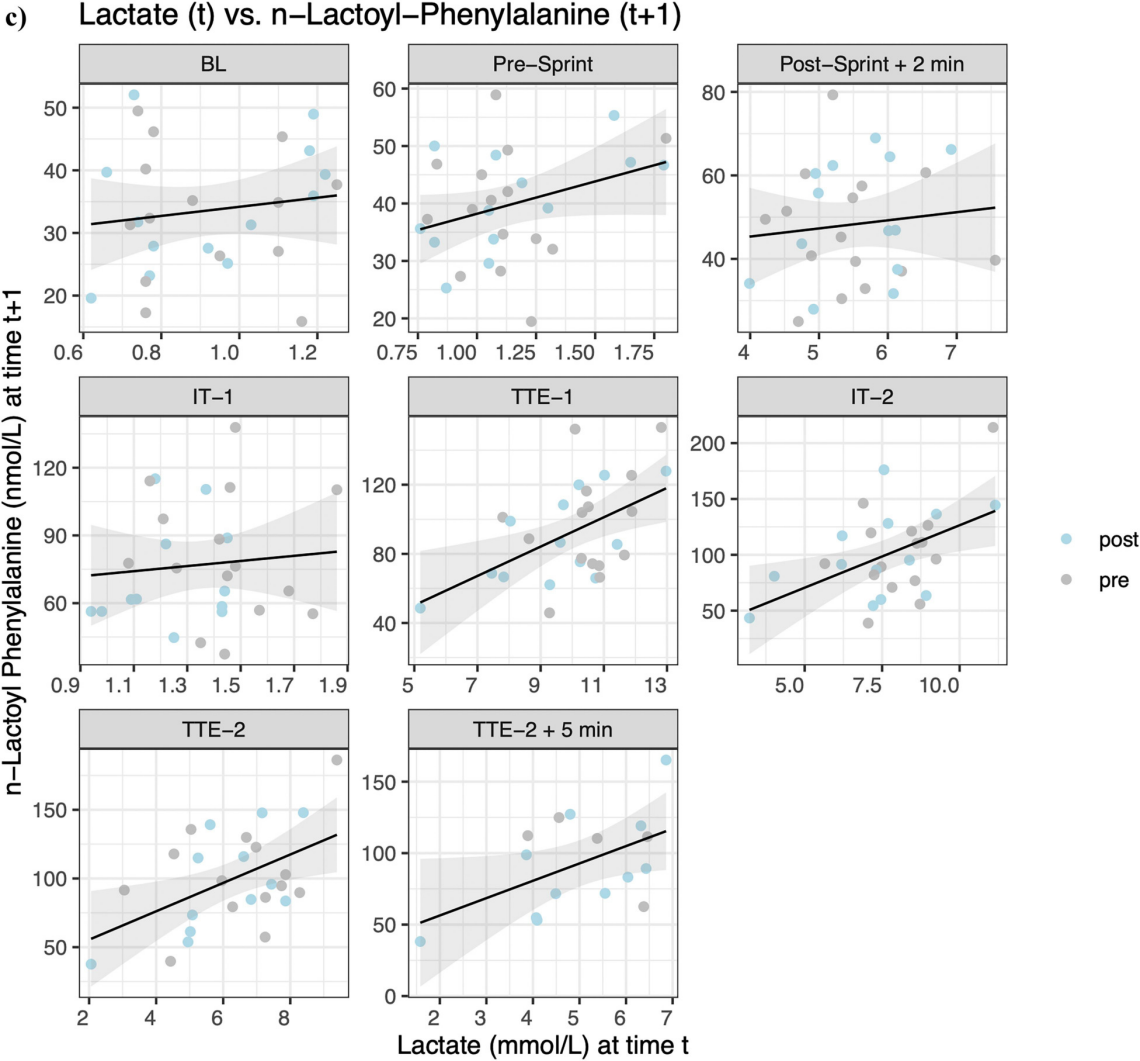


## 4 Discussion

To the best of our knowledge, this is the first study to quantify lac-phe not only before and after, but during exercise providing a more detailed insight into changes across different exercise intensities and modalities. Analysis and quantification were conducted using LC–MS/MS and deuterated internal standard d_5_–lac–phe. The developed and validated method proved suitable for frequent and minimally invasive sample collection (18). Beyond demonstrating proof-of-concept, the study adds to the evidence on lac-phe dynamics during exercise in a group of regional youth squad triathletes. Baseline dbs lac-phe concentration was 31.5 ± 10 nmol/L, increasing with exercise and slightly decreasing within five and 15 min after exercise. Most subjects reached their maximum at time–to–exhaustion after the second incremental test (TTE-2, *n* = 11, 101.0 ± 40.1 nmol/L). In comparison, Li et al. reported plasma lac–phe baseline concentrations of 25.6 ± 7.4 nmol/L and an average maximum of 206.8 ± 62.7 nmol/L after a 3 × 30 s sprint test in a moderately trained men (5). That study showed 1.5–8-fold increase depending on exercise modality, while the present protocol (5), induced a 3-fold increase, comparable to data in obese subjects after a 30-min endurance exercise at 80% V˙O_2_peak (6). Despite differences in nutrition protocols (fasted vs. standardized CHO rich), sampling media (plasma vs. dried blood), and training status (untrained vs. trained), baseline lac-phe values were similar across studies (5), but not comparable to another study due to a lack of quantification of lac-phe (6). The present lac-phe curve suggests a decline within 15 min after exercise, in contrast to the prolonged increase after sprint exercise reported by Li et al. (5). This may relate to different lactate kinetics. Sprints cause higher lactate production than elimination, whereas the present intermittent protocol results in different lactate production and lactate elimination ratios, affecting substrate availability for lac-phe formation (4). The lower lactate concentration after the second incremental test might be explained by different exercise modalities (cycling vs. running) (19, 20) or a substrate shift from CHO to fat oxidation comparing the first and the second incremental test (first incremental test: CHO oxidation: 3.0 ± 0.5 g/min, fat oxidation: 0.1 ± 0.1 g/min; second incremental test: CHO oxidation: 2.8 ± 0.5 g/min, fat oxidation: 0.3 ± 0.2 g/min) (21, 22).

An eight–week training intervention did not affect lac-phe concentrations during the same exercise protocol in a previous study (6), consistent with the present pre-post comparison. While significant baseline/rest-correlations between lactate and lac–phe have been reported (5, 6), this was not confirmed in this study. Including values measured during exercise yielded opposing results, suggesting a distinct physiological regulation of lac-phe and lactate during exercise. Further, no correlations were found between dbs lac–phe concentrations and capillary lactate, capillary glucose, bioimpedance parameters (BMI, MM, FFM, FM, BFP, BW), and spirometric parameters (EEx, CHO oxidation, fat oxidation, $\dot{V}\;O_{2}$max).

In this study, female subjects showed higher lac-phe concentrations than male subjects. However, due to the small sample size and modest effect size, this finding requires confirmation. If lac-phe contributes to post–workout appetite suppression as suggested in men (23), the high increases in female subjects, as shown in the present study, could indicate a similar effect in women. Reported sex differences in muscle glycogen concentration, with females showing lower glycogen concentrations in the gastrocnemius, equal glycogen concentrations in the vastus lateralis, and lower glycogen net utilization during endurance and interval exercises (24), would suggest lower lactate production and thus contrast with the present findings. $\dot{V}\;O_{2}$max differed by sex (males: 63 ± 4 ml/kg/min, females: 54 ± 6 ml/kg/min), consistent with literature showing 20% higher values in men (25, 26). However, when adjusted for lean mass or skeletal muscle mass, these differences diminish or disappear (25, 27), as confirmed here (males: 68.0 ± 5.0 ml/kg FFM/min, females: 66.4 ± 4.3 ml/kg FFM/min), making training status an unlikely explanation for sex-specific lac-phe differences.

### 4.1 Potential role of lac-phe in athlete testing

While overeating is a major contributor to obesity, athletes often face the opposite challenge meeting their high energy demands. Prolonged or frequent appetite suppression resulting in (long-term) reduced energy intake can lead to energy and nutrient deficiency (28). Chronic underfuelling, defined as energy availability below 30 kcal/kg FFM, has physiological and psychological consequences summarised as relative energy deficiency in sports (RED-S) (7, 8). Low energy intake in athletes is often assumed to be present due to tight schedules, lack of nutritional knowledge, body image, disordered eating behaviour, and external and internal pressure (29–31). Although there is recently some doubt about the conceptualisation of RED–S as a syndrome and its cause (32), Brooks et al. state: “Anecdotally, hunger disappeared in studies on 12-h fasted men given exogenous lactate infusion. Of note, athletes competing in 400–1,500 m runs that result in extraordinary lactatemia are seldom hungry immediately after hard training or competition” (33). Exercise–induced appetite suppression is generally assumed to occur at intensities above 60% $\dot{V}\;O_{2}$peak (23). The identification of lac-phe as a potential appetite–suppressing metabolite adds another possible explanation for the underfueling in athletes. Energy homeostasis and therefore energy intake is regulated by a complex interplay of physiological, behavioral, and environmental factors (10, 23). However, the causal relevance for humans remains uncertain. For instance, diet-induced obese mice lost weight after receiving an injection of 50 µg/kg BW lac-phe (5), which increased plasma concentrations more than 100-fold – far above physiological concentrations observed after exercise (34). The gastrointestinal tract alone secretes more than 20 peptide hormones that influence digestion, satiety, and energy balance, many of which are sensitive to nutrient content and regulate short-term hunger (35). The appetite-suppressing mechanism of lac-phe is still unclear. There are some hypotheses on lac-phe affecting the hypothalamus by passing through the blood-brain barrier whose permeability is increased by obesity and exercise and affects G-coupled receptors in the brain (34). As phenylalanine is affecting G-protein coupled receptors, lac-phe might just have a role as a blood-brain barrier crossing metabolite and, once having crossed dissolve into lactate and phenylalanine and might therefore be a factor just enriching cerebral concentrations of lactate and phenylalanine. Identifying the specific receptor(s) and target cell types is therefore essential to understand the potential role in appetite regulation of lac-phe (23). Exercise-induced increases in lactate are also known to influence gut hormones activated by the hypothalamic-pituitar*y* axis, including acylated ghrelin, glucagon-like-peptide-1 (GLP-1), peptide YY (PYY), pancreatic polypeptide (PP) and cholecystokinin (CKK) (9, 10, 23, 33, 34). Previous research also shows suppression of acylated ghrelin and increasing levels of PYY, GLP-1, and PP for 2–9 h after exercise (36, 37). Among these, ghrelin appears to play a particularly pivotal role in mediating lactate-induced appetite suppression, especially within the first within 1–2 h after exercise (9, 10, 38, 39). To clarify lac-phe's relevance for exercise-induced appetite-suppression, and thus its potential contribution to low energy availability in athletes, future studies should assess gut hormone concentrations, feeling of satiety, energy expenditure, and energy intake simultaneously.

## 5 Conclusion

This study is the first study to quantify lac-phe concentration in dried blood spot samples and to describe lac-phe kinetics before, during, and after exercise in healthy and trained regional junior squad athletes. The findings confirm exercise-induced increase in lac-phe consistent with previous observations in mice, racehorses as well as obese and untrained humans (5, 6). Whether physiologically increased lac-phe levels, showing a 3-fold increase in this and other endurance exercise studies, induce appetite suppression in lean humans needs to be investigated. Future research should simultaneously analyze the signaling pathway of lac-phe, target receptors targeted, associated gut hormone concentrations, feeling of satiety, energy expenditure, and energy intake. Should a causal link between lac-phe and appetite suppression be established, lac-phe ought to be acknowledged as a key metabolic factor in RED-S research and athlete health monitoring.

## Data Availability

The original contributions presented in the study are included in the article/Supplementary Material, further inquiries can be directed to the corresponding author.
