# Supplementary material for: Proof-of-concept of a prior validated LC-MS/MS method for detection of N-lactoyl-phenylalanine in dried blood spots before, during and after a performance diagnostic test of junior squad triathletes
Source: Front Sports Act Living. 2025 Sep 30;7:1600714. doi: 10.3389/fspor.2025.1600714 (PMC12518242; doi:10.3389/fspor.2025.1600714)
Supplement: Supplementary file 1 [file Supplementaryfile1.pdf]

Supplementary Material. Dried blood spot analysis of lac-phe before, during and after exercise in junior athletes

1 Development of an analytical method for N-Lactoyl-Phenylalanine (lac-phe) detection by LC-MS/MS

1.1 Reagents and Chemicals

For volumetric collection, 20 µL volume-absorbing end-to-end capillaries coated with K2E were used obtained from EKF diagnostics (Barleben, Germany). Cellulose-based dbs cards (QIAcard FTA DMPK-C) and MiniPax absorbent packs were purchased from Sigma Aldrich (Taufkirchen, Germany). Acetonitrile (ACN), methanol (MeOH) and formic acid (FA) were obtained from VWR chemicals (Langenfeld, Germany). Human serum albumin (hsa) (5 %) was obtained from Biotest Pharma GmbH (Dreieich, Germany). N-lactoyl-phenylalanine (lac-phe) was obtained from Merck (Germany). Ultrapure water was received from a Barnstead GenPure xCAD Plus from Thermo Scientific (Bremen, Germany). To quantify lac-phe in dried blood spots (dbs), a deuterated internal standard d5-lactoyl-phenylalanine was synthesized in the laboratory of the Institute of Biochemistry.

1.2 Synthesis of (2-hydroxypropanoyl) (2,3,4,5,6 d)-phenylalanine (d5-LacPhe) and working solution

(2,3,4,5,6 d)-phenylalanine (170 mg; 1.0 mmol; 1 eq) was placed in a flask and was dissolved in H_2O (162 mg; 9 mmol; 9 eq). To this solution, lactic acid (600 µL; 8 mmol; 8 eq), and calcium oxide (16.6 mg; 0.3 mmol; 0.3 eq) were added. The mixture was then stirred for 2 h at 100 °C. After cooling to room temperature, was worked up as follows.

A chromabond solid-phase-extraction-cartridge (C18 SPE; 6cc) was conditioned with MeOH (3 mL) and H_2O (3 mL). The reaction mixture was added to SPE and then eluted with an ACN/H_2O gradient (10 % to 60 % ACN). Following this, the solvent was removed under reduced pressure and the desired

product d5-lac-phe (0.13 g; 53 %) was isolated as a white solid. Internal standard (ISTD) stock solution was prepared in 10 % ACN and deionized water at 10 ng/mL [12]. The stock solution was stored at -20 °C.

1.3 Liquid chromatography high resolution mass spectrometry

For mass spectrometric characterisation of the standard compounds lac-phe and its deuterated analog an LC-ESI-HRMS/MS system by Agilent (Santa Clara, California, USA) was used. For gradient elution, 0.1% FA in H2O was used as Eluent A and 0.1% FA in ACN was used as Eluent B. The gradient started with 0% B, increasing to 100% within 4 min where it was held for 3 min. After returning to starting conditions within 0.01 min, the column was re-equilibrated for 3 min. A flow of 0.3 mL/min was applied. The injection volume was 5 µL.

MS/MS Data were collected using a dual AJS ESI source in negative ionization mode with an ionization voltage of -4000 V and a sheath gas temperature of 400 °C. The parent mass for MS2 experiments were extracted with a width of 1.3 m/z and both, 25 and 35 were used for collision energy.

1.4 Sample Preparation

The whole punch of the dbs card (20 µL of sample) was extracted into 600 µL of MeOH and 10 µL of d5-lac-phe (c = 0.001 mg/mL) in an 1.5 mL Eppendorf tube, subjected to ultrasonication for 30 min. Afterwards, the same Eppendorf tube was centrifuged at 17,000 × *g* and 19 °C for 10 min. The supernatant was transferred into a new 1.5 mL Eppendorf tube and evaporated using a vacuum centrifuge at a temperature of 45 °C for 90 min. The resulting dry residue was reconstituted in 100 µl

of 10 % ACN solution in a further new 1.5 mL Eppendorf tube and centrifuged at 17,000 × *g* and 19 °C for 10 min. The supernatant was transferred into vials and analyzed by LC-MS/MS.

1.5 Liquid chromatography tandem mass spectrometry

LC-MS/MS analysis of dbs samples was conducted using an ACQUITY I-Class ultra-performance liquid chromatograph (UPLC) coupled to a Xevo Triple Quadrupole-XS mass spectrometer (TQ-XS-MS), both from Waters (Eschborn, Germany). Samples prepared for analysis were injected onto a Poroshell C-8 analytical column ((50 × 3.0 mm, 2.7 µm particle size); Agilent, Waldbronn, Germany) using the eluents A (0.1 % FA in H_2O) and B (0.1 % FA in ACN) at a flow rate of 0.3 mL/min. The method was started with 0 % B increasing to 100 % B within 8 min followed by equilibration at starting conditions for 3 min. The overall runtime was 11 min. The compounds were introduced into the mass spectrometer by electrospray ionisation (negative mode) and were detected by time-based multiple-reaction monitoring. Predicted retention time was set at 3.30±0.5 min. Cone voltage was 22 V and collision energy 15 eV. The data were processed using TargetLynx (Waters, Eschborn, Germany). Product ions at m/z = 88 and m/z = 91 were used for lac-phe quantification (s. Figure S2a, Figure S2b).

1.6 Method validation

Analytical method validation was performed following the main requirements of the U.S. Food and Drug Administration (FDA) guidelines for bioanalytical method validation [11]. These include parameters like linearity, precision, accuracy, carryover, matrix effects, recovery, selectivity, stability, limit of detection (LOD) and limit of quantification (LOQ).

2 Results

Chromatograms of all analytes of a blank which was 5 % hsa (a), a calibrator level in the middle of the working range of 40 ng/mL (b) and an authentic sample is shown in Figure S3.

A summary of all the validation results is presented in Table S1. Calibration curves were found to be linear over the ranges of eight individual calibrator concentrations, with R^2 greater than 0.99. Additionally, the precision and accuracy results met the FDA criteria, as the variation coefficient is below 15 %. Carryover effects are below 1 %. Matrix effect is 187 %, which indicates a matrix-based enhancement. The recovery was 72 % for a 100 ng/mL sample and 101 % for a 5 ng/mL sample. In terms of selectivity, the eight samples displayed no interfering signals at the expected retention times of the analytes, owing to sufficient chromatographic separation. Stability is confirmed for more than 28 days for both storing temperatures room temperature and -20 °C. The method’s limit of quantification (LOQ) and the limit of detection (LOD) were found to be suitable for assessing the levels of endogenous lac-phe.

List of supplemental figures and tables

Figure S1 Individual concentration of a) glucose, b) lactate and c) lac-phe over the course of the protocol.

a)

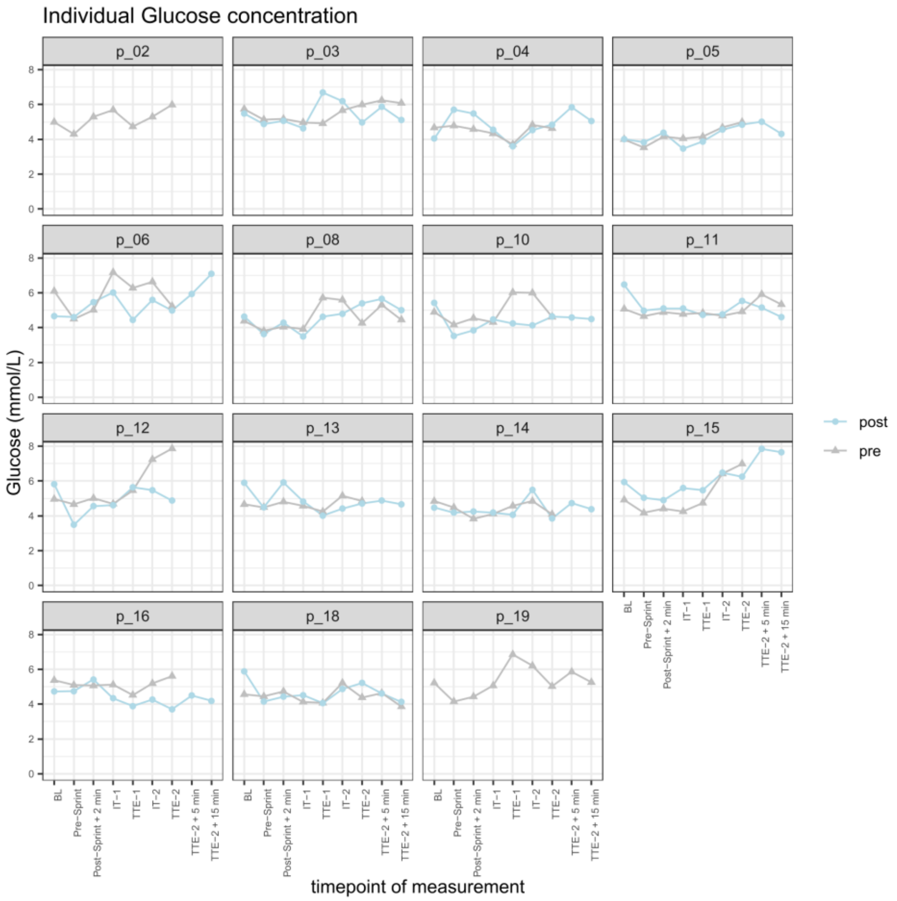

b)

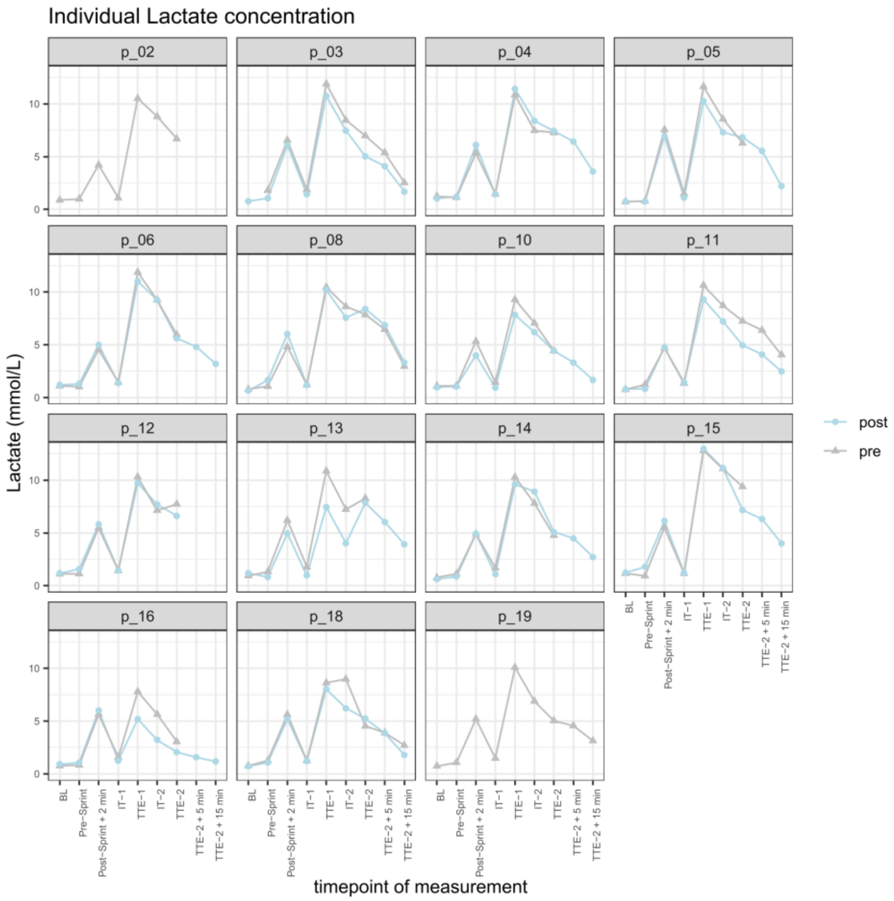
Individual Lactate concentration
p_02
p_03
p_04
p_05
p_06
p_08
p_10
p_11
p_12
p_13
p_14
p_15
p_16
p_18
p_19
Lactate (mmol/L)
0
5
10
post
pre
BL
Pre−Sprint
Post−Sprint + 2 min
IT−1
TTE−1
IT−2
TTE−2
TTE−2 + 5 min
TTE−2 + 15 min
timepoint of measurement

c)

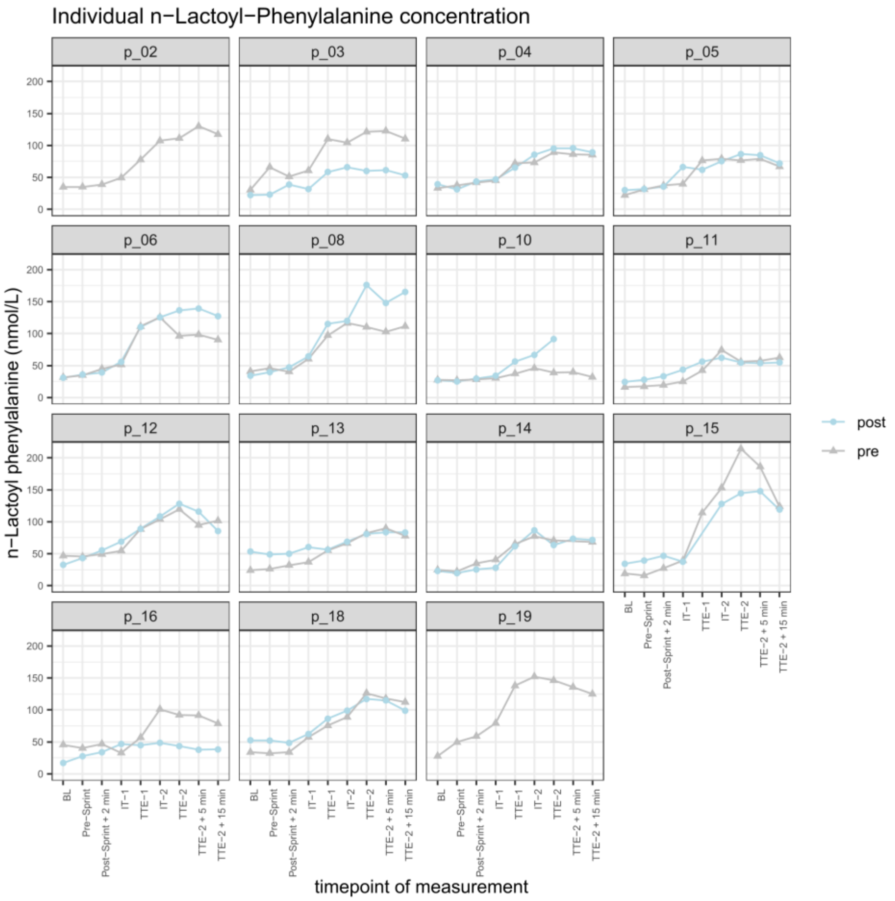

Individual n−Lactoyl−Phenylalanine concentration
p_02
p_03
p_04
p_05
p_06
p_08
p_10
p_11
p_12
p_13
p_14
p_15
p_16
p_18
p_19
n−Lactoyl phenylalanine (nmol/L)
timepoint of measurement
post
pre
BL
Pre−Sprint
Post−Sprint + 2 min
IT−1
TTE−1
IT−2
TTE−2
TTE−2 + 5 min
TTE−2 + 15 min

Figure S2 ESI-product in mass spectra for (a) *N*-Lactoyl-Phenylalanine (lac-phe) ([M-H]$^-$ = 236) and (b) d5-Lactoyl-Phenylalanine (lac-phe) ([d5-M-H]$^-$ = 241) recorded using a Agilent 6546 LC-Q-TOF (Waldbronn, Germany) high resolution tandem mass spectrometer operating in negative product ion mode.

a)

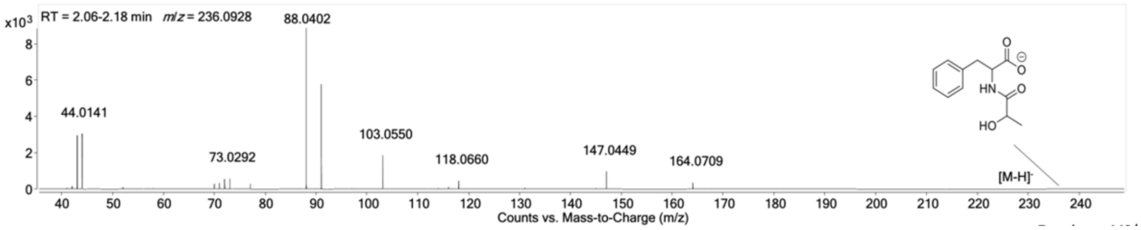

b)

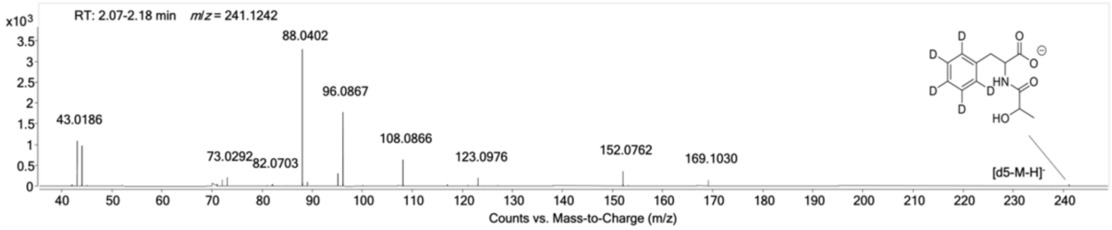

Figure S3 Extracted-ion chromatograms of (a) a negative quality control (QC) which is blank human serum albumin (hsa) (5 %), (b) a positive QC which was 5 % hsa containing *N*-Lactoyl-Phenylalanine (lac-phe) in a concentration of 40 ng/mL and (c) an authentic dried blood spot sample lac-phe metabolites.

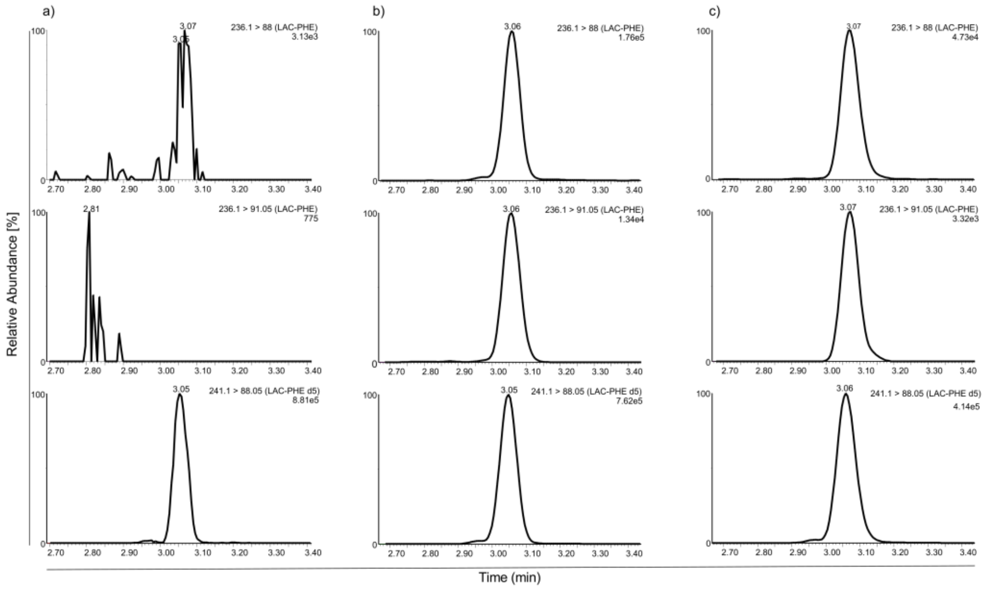

Table S1 Results of the validation of *N*-Lactoyl-Phenylalanine (lac-phe) according to requirements of the U.S. Food and Drug Administration (FDA) guidelines for bioanalytical method validation [32] within a working range of 0-100 ng/mL.

Validation parameter	Concentration of lac-phe in 5 % hsa (ng/mL; nmol/L)	Result
Linearity (R^2)	0-100; 421.5	0.9976, y = 0.0059x – 0.0039
Precision (n = 6)		
	5; 21.1	3%
	100; 421.5	8%
Accuracy (n = 6)		
	5; 21.1	107%
	100; 421.5	95%
Carryover	100; 421.5	<1%
Matrix effect	20; 84.3	187% (enhancement by matrix)
Recovery (n = 6)		
	5; 21.1	101%
	100; 421.5	72%
Selectivity (n = 8)		No interfering signals
Stability (n = 22)		
	100; 421.5	Stable for at least 28 days at -20°C
	100; 421.5	Stable for at least 28 days at room temperature
LOD (n= 8)	S/N ≥ 3: 1 ng/mL	Estimated at *m/z* = 88 and *m/z* = 91
LOQ	S/N ≥ 9: 3 ng/mL	Estimated at *m/z* = 88 and *m/z* = 91
